# A Health Education Website Developed to Meet Young People’s Information Needs About Web-Based Pornography and Sharing of Sexually Explicit Imagery (SCOPE): Usability Study

**DOI:** 10.2196/12824

**Published:** 2019-08-13

**Authors:** Angela C Davis, Cassandra J C Wright, Meredith J Temple-Smith, Margaret E Hellard, Megan S C Lim

**Affiliations:** 1 Burnet Institute Melbourne Australia; 2 School of Public Health and Preventive Medicine Monash University Melbourne Australia; 3 Department of General Practice Melbourne Medical School The University of Melbourne Melbourne Australia

**Keywords:** adolescent, pornography, health promotion, internet, sex education

## Abstract

**Background:**

Although we know that many young people watch online pornography and engage in *sexting*, there is limited literature examining their needs in relation to information on these highly sensitive and complex issues. Online resources exist; however, we can find no evidence of any of them having been formally tested for usability within the target population.

**Objective:**

This study aimed to test the usability of a resource about online pornography and sexting among young people.

**Methods:**

Semistructured interviews were conducted with 17 participants aged 15 to 29 years.

**Results:**

We found that the *SCOPE* resource was perceived as trustworthy and credible because of its evidence-based content, nonjudgmental tone, and balanced perspectives. Multimedia and video content enhanced the layout and usability of the resource; however, content relevance could be improved by targeting age and developmental stages. Participants identified resource sections such as *Real Stories* from young people as relevant and engaging. However, they raised issues with the translation of formative research findings relating to these stories into their final presentation.

**Conclusions:**

Our findings suggest that young people prefer online resources about complex issues, such as online pornography and sexting, if they are balanced in content and tone. Most importantly, in the context of responding to complex and sensitive issues such as these, co-design methods can ensure that young people are central to the development of resources and avoid gaps in translating research into practice. In the context of limited literature focusing on the usability of online resources about these topics, this paper provides important insights for public health practitioners working in this emerging space.

## Introduction

### Background

Researchers and communities have raised concerns about the high prevalence of exposure to online pornography among young people [1] as well as the relatively high incidence of sharing of personal sexually explicit imagery (sending nudes or sexting) [2]. Although public health research in this area is increasing [1,2], evidence about the effects of engagement with new sexual media remains mixed [3]. Studies have shown that young people are frequently exposed to sexual risk behaviors, gender stereotypes, and inequality when they view pornography [4]. Viewing pornography has been shown to be associated with early age of first sex, condomless sex, sexist attitudes, and aggression [3]. For example, a large (n=4564) survey of young people aged 14 to 17 years found that boys who regularly watch online pornography were significantly more likely to express negative gender attitudes [5]. Studies are yet to describe the direction or strength of these associations to be able to identify a causal relationship between pornography and harm [6,7].

Some literature even suggests that young people may experience pornography as an important source of education and exploration in the context of limited alternative education about sex [8,9]. Similarly, while sharing sexually explicit images, sometimes referred to as *sexting* or *sending nudes*, has been shown to be common among young people [10,11] and nonconsensual sharing or technology-facilitated violence has been shown to have damaging health consequences [12], some studies suggest that young people may see consensual sharing of images as a normal and even positive part of their sexual lives rather than as inherently risky behavior [2,13]. Pornography and sexting are complex and sensitive topics with limited consensus on the best practice for researching and responding to the issues [6,14]. Although the effects are still being investigated, experts and young people have expressed a need for specific online health resources to address unmet education needs around these topics [15].

Extensive literature has shown that online health promotion approaches are most successful if they are tailored, nonjudgmental, and responsive to young people’s information needs [16]. Engaging with target users in the development and testing of resources can enhance the relevance of the resource [16,17]. In terms of usability, studies also suggest that young people prefer interactive content, opportunities to engage with other young people, and easy-to-navigate resources without too much text [18]. Although a large body of evaluative studies of sexual health websites exists [19], few studies have specifically evaluated the relevance and usability of health websites addressing pornography and the sharing of sexually explicit media images for young people. This is an important step in the development process for evidence-based resources.

### SCOPE Website Development

**Figure 1:**
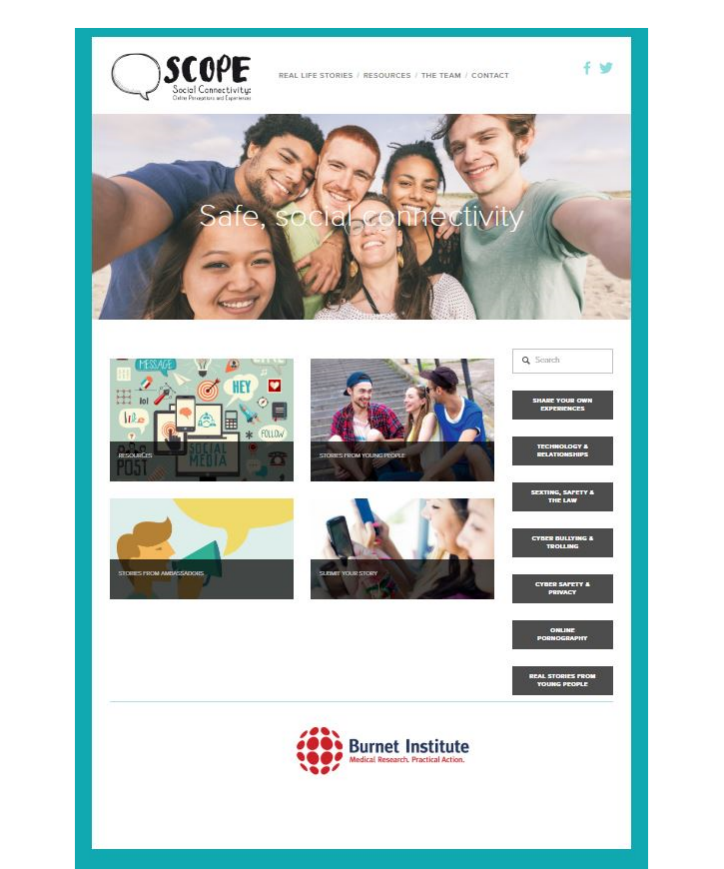
SCOPE Online Resource home page.

In late 2015, the Burnet Institute received a small grant from a philanthropic foundation to develop an online resource focused on technology and relationships. Consistent with the best practice health promotion theory, we conducted formative research with a target audience of young people aged 15 to 29 years from Australia [14]. The findings of the research led us to develop a website, predominantly focused on the issues of managing personal sexually explicit imagery, pornography use, and general online issues, including cyberbullying (Figure 1). The website included 3 sections: an information page on each of the topics listed above, a resources section with links to services and other sites, and a real stories page with user-generated content about the issues. On the basis of participant feedback, we used sex-positive, nonjudgmental language and integrated user-generated stories from young people relevant to the key topics to enhance the credibility and relevance of the information. We also provided information about the services and laws relevant to the topics (ie, laws related to distributing personal sexually explicit imagery in Australia) [14]. The website was developed as a minimum viable product with which to conduct usability and content testing with the target audience before further implementation and evaluation. After the website was developed, we planned a small formative evaluation to test the website’s usability, relevance, and acceptability before any scale up.

### This Study

This study investigated the relevance, usability, and acceptability of the SCOPE website among its target audience. Insights are intended to inform development of future online health promotion interventions, with particular application to websites and resources about sexually explicit media and young people.

## Methods

### Participants

The eligible participants were aged 15 to 29 years and living in Australia.

### Recruitment

Convenience sampling methods guided our recruitment of participants using paid advertisements on Facebook and a post from the Burnet Institute Facebook account. Potential participants were invited to contact the research team via an online contact form or email if interested. Participants were told that the interview involved exploring a website and talking to researchers about their opinions and feedback as they explored the content. They were advised that the interviewers were not involved in the website development. The Monash University Human Research Ethics Committee approved the research, and participants consented to the publication of anonymized data.

### Procedure

Following consent, participants were sent a unique link to access a private, secure chat room where they *met* the researcher at a prearranged time for the interview. Participants were invited to take part in one-on-one, semistructured interviews through Atlassian HipChat, an online text-based chatroom tool. Interviews involved the researcher guiding the participant through predefined sections of the website and asking semistructured interview questions about each aspect of the website usability, including layout, language, platform, and content relevance. The participants were specifically directed to access the home page, real stories page, online pornography page, and *sexting* pages of the website. They were asked to explore and read the content and alert the interviewer when they were ready to discuss each section. Interviews ranged from 45 to 60 min, and the participants were reimbursed with a Aus $20 gift voucher.

The rationale for using online interview methods included that we wanted to engage with participants in real time as they explored the resource to capture their thoughts and opinions. We also wanted to guide them through specific content on the website and ask relevant questions immediately after they had engaged with the section and to reduce social desirability bias. Using a traditional postexposure survey would not have enabled this type of engagement. Furthermore, we were concerned that an in-person guided exposure and face-to-face interview may not allow participants to be open and honest about these sensitive issues or offer their opinions. Young people are digital natives who communicate frequently via online platforms. In this context, conducting qualitative interviews in private chatrooms has previously been shown to be an acceptable and convenient method of eliciting meaningful data on sensitive topics from young people, with some limitations [20-24]. For example, conducting online interviews with young people can increase their likelihood of signing up; however, building a rapport can be more difficult, and participants can withdraw from the interview simply by not answering. [20]. Despite these limitations, the method is particularly effective for testing website content because participants have a level of anonymity and separation from researchers, allowing them to explore the website in an organic setting (their own device and at their own pace) and to be open and honest about sensitive topics such as pornography.

### Data Analysis

Deidentified transcripts were analyzed thematically using QSR International NVivo software. Framework analysis methodology guided a deductive thematic analysis, and additional themes were inductively coded as they emerged [25]. Framework analysis has been identified as an ideal qualitative method for interpreting data specific to public health policy or practice [25]. The analytical framework for this study was refined by AD based on the process of familiarization, which involves a preliminary review of the entire dataset in relation to answering the key research question: *How does the target audience perceive the usability of the online resource?* The framework included 3 key nodes: relevance, usability, and acceptability. Each included 2 subnodes: barriers and enablers. Researcher AD deductively coded the entire dataset. Subthemes were inductively identified as they emerged across each of the nodes. Researcher CW cross coded a sample of transcripts. Discrepancies in interpretation or coding were discussed and resolved before finalization of analysis.

## Results

### Participant Characteristics

A total of 17 young people participated in the interviews. Participants’ age range was 15 to 27 years with an average age of 21 years. Overall, 15 participants identified as female and 2 identified as male. All participants were living in the greater Melbourne region. In total, 3 participants were still attending secondary school, 10 were university undergraduate students, and 4 had completed a bachelor’s degree or postgraduate degree.

### Resource Usability

The 3 themes identified across the data relating to the resources usability are reported below and include *responding to sensitive health issues*, *translating formative findings into acceptable content,* and *who are young people?—creating acceptable and accessible content*.

#### Responding to Sensitive Issues: Balance, Tone, and Credibility

Creating content and a tone that is responsive to the sensitive and complex nature of these issues was perceived as the most important aspect of the SCOPE website’s acceptability. For instance, information about pornography and sharing of sexually explicit imagery was identified as *balanced* and nonjudgmental. This was perceived to be in contrast to fear-based or values-based resources on issues such as pornography and sharing of sexually explicit images. As participants stated:

Some [websites] are very obviously politically motivated or have some sort of agenda, and provide either false facts or twisted facts to suit their message. Some websites can present things in an “objective” way but obscure the context to fit their politics—it can take more skill and digging to figure this out. I think being aware of a website's agenda is something useful when gathering info.

P11

As the responses demonstrate, participants were critical of other resources perceived to be judgmental or motivated by social or political agendas. There was a sense of relief that SCOPE acknowledged that many people use pornography and that this was not negatively framed:

The fact that they mention that porn isn't a bad thing is a breath of fresh air since growing up porn has been viewed as a bad thing...it doesn’t sound cheesy or lecture-y.

P14

On the basis of their concerns about biased or values-based information, several participants mentioned the importance of credibility. One feature of the SCOPE website that participants noted made it appear *trustworthy* was the use of linked citations to substantiate information and the provision of secondary research related to the topics. However, research links were not provided for every piece of information across the sections. In the context of sensitive and politicized issues, providing direct access to research across all content would improve acceptability as this participant stated:

The first thing I wanted to read is the research it mentions—it’d be good to have a link to that (sorry I’m so obsessed with credibility!!).

P3

**Figure 2:**
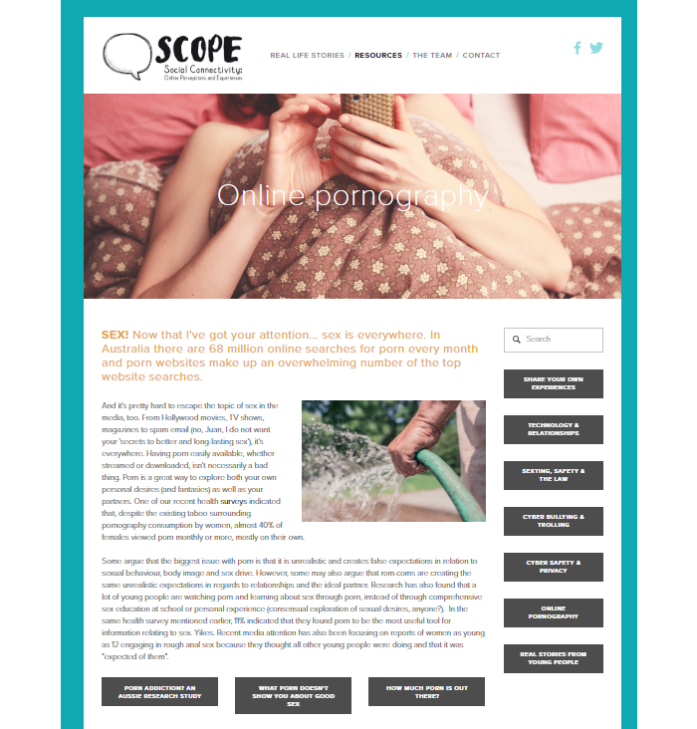
SCOPE Online Resource–Online Pornography page.

The website was perceived to be visually responsive to the sensitive nature of the issues. For instance, images were suggestive of the topic but displayed a level of subtlety. For example, as shown in Figure 2 below, the Online Pornography page images show a person on a phone in bed and a water hose. These are suggestive—without showing explicit images. This enabled participants with various experiences and backgrounds to engage without feeling uncomfortable. As this participant commented:

I like the top photo, it’s not too revealing, even the wording is subtle which is good for reading in public

P14

Participants appeared conscious of their positioning as *young people* in relation to the tone and language of the resource. According to the participants, the language used throughout the website was clear and accessible without being *childish* or *patronizing*. This is explained here by a participant who said:

It’s very suited to young people. It’s easy to understand, but a step up from language you would use with a child.

P4

#### Translating Formative Findings Into Acceptable Content

**Figure 3:**
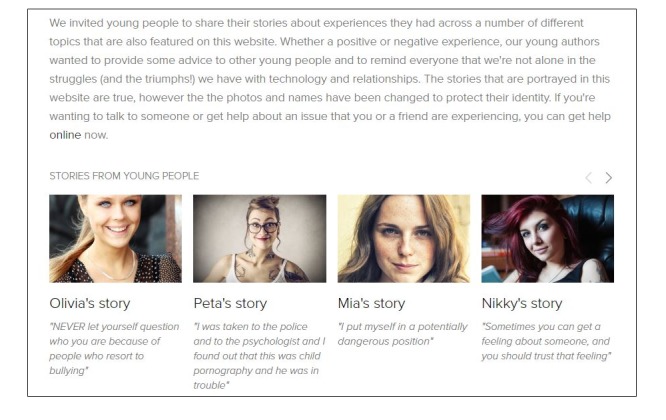
SCOPE Online Resource–Real Stories page.

On the basis of findings from formative research, the Real Stories page illustrated in Figure 3 was marketed as user-generated content about young people’s experiences with technology. Young people were invited to send in their stories via the website or social media. The aim of the content was to reduce shame about negative experiences and encourage awareness among other young people. In this study, we wanted to understand if the Real Stories section was usable and acceptable. Our participants reported that including real stories from other young people helped to acknowledge the complexity of the issues. Participants appreciated that the stories did not attempt to simplify the issues by acknowledging the complexity of young people’s online experiences. The stories raised issues as part of a broader discussion of harm reduction rather than as warning or moralistic messages. Their comments also reflected a sense of frustration around advice they had encountered elsewhere to restrict online use by *logging off as* a response to these concerns. As this participant suggested:

The story was good because it wasn’t just black and white, like it’s a hard decision to make and it’s not that easy just logging off.

P14

Although the website presented stories from young people that were real, stock images were used in place of an actual biographical profile picture to protect the anonymity of young people who shared their stories. A number of participants also raised concerns about the page because they felt it was unclear if the stories and people were real, which reduced their trust in the content overall. Participants’ critical media literacy reduced their acceptance of the Real Stories page, which was a key aspect of the website. As this participant stated:

I just clicked through to one though and it was like a cautionary tale of sharing photos on the internet with people you haven’t met. It uses a stock image photo next to the story, so I’m not sure if it is real? Young people are very aware of reverse-image searching on Google. All of them use Shutterstock photos, which shows like Catfish teach young people how to discern pretty quickly (sic). And also the site links to Catfish itself, so it seems a bit of a contradiction...So any story about a young person that purports to be real, alongside a photo that’s a stock image, may not be successful...I am skeptical of the website being helpful for info if the stories are not genuine.

P1

Despite their concerns about the presentation of Real Stories, most considered including *real stories* from young people as enhancing the acceptability and relevance of the resource. However, many identified barriers to actually sharing their own stories on platforms such as this. These included, being retraumatized by telling their story and experiencing embarrassment—regardless of the website’s commitment to anonymity. Participants reported that they would also be unlikely to share the content on social media between friends because of the sensitive nature of the topics. As these participants reflected:

I think it can be very embarrassing and I would always have the fear that someone could identify me through the story. It can also be a very sensitive topic and people might not be confident/ready to share with the world.

P2

I suppose anonymity and embarrassment! I don’t think any young person wants everyone at school or community to know their embarrassing story. That’s why so many of these stories are told in hindsight, which is a shame cause teenagers could probably benefit from knowing they’re not alone from their own peers rather than adults. That said, I wouldn’t feel comfortable submitting my story because I’m naturally shy, and I also generally worry about safety and so on.

P11

Participant responses to the section highlight potential challenges to translating users’ needs and wants into relevant and acceptable content. Participants’ concerns about the disingenuous nature of the real stories content because of the use of stock images and their own reservations around sharing personal stories highlight potential impacts on audience engagement when an idea developed in formative research stages does not fully translate into acceptable content.

#### Who Are Young People? Creating Acceptable and Accessible Content

To understand the acceptability of the website, we asked participants who they thought it was aimed at and who would use it. Although some participants saw the website as personally relevant, many identified people *younger* than them as the intended audience. They perceived groups of *preteen* young people, as more naive, less experienced, and in need of this type of information, as is illustrated in the exchange below. Others said that the website looked like something “my mother would send to me” (P7), which suggested that adults might be more concerned about these issues than young people themselves:

Do you think that this information would be relevant for young people?

Interviewer

Yeah young people, probably pre-teen like year 7-10 (sic).

P14

Ok, why that age group?

Interviewer

because we’re already aware of these issues, some of use face them on a daily basis, or at least hear about them. Like personally it’s common-sense—some of these issues you should know what you should and shouldn’t do (sic).

P14

As savvy users of online content, our users were highly critical of accessibility of the website. Although the home page layout supported easy navigation, the online pornography and sexting pages could be improved through interactivity, links, and videos at the top of the page and by reducing the amount of text. As participants explained:

I’m sceptical that young people would read that much text so maybe the images/buttons/videos could be moved to break it up into smaller bits.

P1

I think the paragraphs need to be broken up using maybe subtitles? (sic) Just to make specific information easy to access.

P2

It still seems like a bit of a lecture—maybe make it interactive and fun, that is, competitions/videos/etc the personal stories would probably be the best though—more relatable.

P14

## Discussion

### Principal Findings

The overall results of our interviews suggest that the resource was perceived as trustworthy and credible because of its evidence-based content, nonjudgmental tone, and balanced perspectives. The resource was also perceived as relevant overall; however, there was a perception that the topics included were more relevant to younger participants and that the Real Stories section was disingenuous. The usability of the resource could be improved by targeting content by age and developmental stages, reduced text, headlines to communicate key points, and increased audiovisual assets. Although the conceptual framework underpinning the resource was perceived as usable, results suggest that issues in the final design of the resource could be improved to enhance overall efficacy.

Respondents were aware of the polarized nature of pornography and sexting and were skeptical of information provided without evidence or balanced narratives. This is consistent with research that suggests that young people have an interest in sexuality and respond best to environments that encourage trust and openness and rely on evidence rather than moralistic undertones [26]. However, relying solely on evidence is challenging when dealing with emerging public health issues such as online pornography, where evidence is still being developed [7]. Our results suggest that this may be overcome by providing young people with the best available information, including on why there is lack a of consensus in evidence, and providing opposing views and discussion points to help them make their own informed decisions.

Potentially overexposing young people to information is often perceived as a barrier to providing sexual health education [27]. This has been shown to be of particular concern in relation to online pornography, as adults fear conversations or information that may encourage their child to seek out the content [28]. Using informative headlines with expandable information sections (*progressive disclosure*) could mitigate this risk, while engaging audiences with various developmental stages and information needs. Presenting information in this way would simultaneously meet the needs of young audiences by decluttering and simplifying information for quick and easy access.

Importantly, the findings remind us that although young people share commonalities, they are not a homogenous group, and this should be considered in the development of health education resources. Furthermore, although the target audience for the SCOPE resource was Australians aged 15 to 29 years, respondents often referred to the importance of this type of resource for younger ages. This could be explained by the literature that suggests that young people typically underestimate the impact of pornography on themselves compared with other people [29]. However, it could also be the case that as respondents pointed out, younger children (10-14 years) are engaging in these behaviors or exposed to the content with little support. This explanation supports the need for research to consider how to develop resources that sensitively meet the information needs of younger children on these topics.

Interestingly, our results demonstrated gaps in translating findings from the participatory development research into the resource. For example, a key finding from the development research was that young people wanted to engage with *real stories* to learn from other young people’s experience [14]. However, in practice, the translation of these findings into a *Real Stories* section resulted in concerns about credibility. Furthermore, our participants identified a range of barriers to submitting their own stories. Responses indicated practical guidance for how to improve credibility, such as not using stock images of young people to illustrate anonymous stories. Participants’ skepticism highlights broader issues with traditional research methods for intervention design, including phased research that involves audiences in formative design and content testing rather than as co-designers of the materials throughout the process. Our findings support the move toward iterative co-design methodologies that engage audiences as experts or *co-designers* to ensure the conceptualization of issues, ideation of approaches to address them, and design of resources that align with their needs and wants [30]. These methods are particularly relevant to developing interventions on topics which are sensitive, complex, and still emerging such as online pornography and sharing of sexually explicit images.

### Limitations

Our study has several limitations. Our recruitment strategy utilized social media advertising, meaning that we attracted those with social media experience and potentially those interested in the topic; it is possible that these perspectives vary from those who do not use social media but who are still at risk of other technology-related harms. Our sample was heavily skewed toward female participants. This is consistent with sexual health research more broadly, where engagement with male respondents has been challenging [4,31,32]. Interviews were conducted online rather than in person, excluding the opportunity to observe participants’ body language and tone. Despite these limitations, the data collection method appeared to provide opportunities for participants to engage with the topics and the content in an anonymous forum. The insights provided suggest that the methods are useful for conducting usability testing of health education resources, particularly those aiming to reach young people, and thus provides important insights relevant to an emerging area of health promotion research and practice.

### Conclusions

A range of service providers and organizations are developing resources to respond to growing concerns of the impacts of new sexual media—such as pornography and sharing of sexually explicit images among young people. However, within resource-constrained environments, content testing for usability, relevance, and acceptability are not frequently conducted. Small-scale studies testing websites before scale-up activities can inform future research and development. Our study illustrates the importance of providing balanced and nonjudgmental perspectives when engaging with young people on these issues. However, it also shows limitations to formative research translation and creating content aimed at all young people rather than diverse groups. Most importantly, in the context of responding to emerging, complex, and sensitive issues, co-design methods can ensure that young people are central to the development of resources to meet their needs and avoid gaps in the context of emerging evidence and practice.
